# Andrographolide Enhances Proliferation and Prevents Dedifferentiation of Rabbit Articular Chondrocytes: An *In Vitro* Study

**DOI:** 10.1155/2015/984850

**Published:** 2015-02-23

**Authors:** Li-ke Luo, Qing-jun Wei, Lei Liu, Li Zheng, Jin-min Zhao

**Affiliations:** ^1^Department of Orthopedic Trauma and Hand Surgery, The First Affiliated Hospital of Guangxi Medical University, Nanning, Guangxi 530021, China; ^2^Guangxi Key Laboratory of Regenerative Medicine, Guangxi Medical University, Nanning, Guangxi 530021, China; ^3^The Medical and Scientific Research Center, Guangxi Medical University, Nanning, Guangxi 530021, China

## Abstract

As the main active constituent of* Andrographis paniculata* that was applied in treatment of many diseases including inflammation in ancient China, andrographolide (ANDRO) was found to facilitate reduction of edema and analgesia in arthritis. This suggested that ANDRO may be promising anti-inflammatory agent to relieve destruction and degeneration of cartilage after inflammation. In this study, the effect of ANDRO on rabbit articular chondrocytes* in vitro* was investigated. Results showed that not more than 8 *μ*M ANDRO did no harm to chondrocytes (*P* < 0.05). DNA content and glycosaminoglycan (GAG) /DNA were, respectively, improved in ANDRO groups comparing to the control (*P* < 0.05). ANDRO could promote expression of aggrecan, collagen II, and Sox9 genes while downregulating expression of collagen I gene (*P* < 0.05). Furthermore, hypertrophy that may result in chondrocyte ossification could not be detected in all groups (*P* > 0.05). The viability assay, hematoxylin-eosin, safranin O, and immunohistochemical staining also showed better performances in ANDRO groups. As to the doses, 3 *μ*M ANDRO showed the best performance. The results indicate that ANDRO can accelerate proliferation of rabbit articular chondrocytes* in vitro* and meanwhile maintain the phenotype, which may provide valuable references for further exploration on arthritis.

## 1. Introduction

Due to poor regenerative capacity, articular cartilage tends to be structural breakdown or degeneration because of diseases, aging, or trauma and finally evolves to osteoarthritis (OA) with poor prognosis [1–3]. Over the course of OA development, catabolic factors such as proinflammatory cytokines are activated, which induces the gradual self-destruction of cartilage coupled with the curb of differentiation of chondrocytes [4–6]. Accompanied with this process is the invasion of non-cartilage-specific extracellular matrix (ECM) with inferior mechanical properties that is produced by dedifferentiated chondrocytes and can prevent chondroprogenitors from remodeling cartilage defects through migration [7–9]. All these contribute to the acceleration of OA. To find an effective anti-inflammatory agent accompanied with the role of inhibiting chondrocytes from dedifferentiation, namely, maintaining the phenotype of chondrocytes, is of significance.

As traditional Chinese medicine since ancient times,* Andrographis paniculata* has been widely used in treatment of various diseases including inflammation and tumors [10]. Evidences of association of* Andrographis paniculata* with skeletal system including arthritis were also found in recent studies [11, 12]. Extract of* A. paniculata* has been shown to affiliate reduction of edema and analgesia in arthritis [11]. It was also demonstrated to prevent osteoclastic bone loss associated with bone metastasis of cancer [13, 14]. Andrographolide (ANDRO) is the main active constituent of* A. paniculata* [12, 15]. ANDRO and its derivatives, a group of diterpenes, have been reported to relieve symptoms of rheumatoid arthritis in a random controlled trial [11]. The anti-inflammatory role of ANDRO has been well documented in several studies [16, 17]. Andrographolide also has proapoptotic effect on tumor cells [18, 19]. On the other hand, it was proven that ANDRO facilitated cell differentiation [20]. These findings suggested that as potent anti-inflammatory agent, ANDRO may exert an effect on chondrocyte differentiation, which is a vital part in treatment of arthritis in the long run.

Based on the hypothesis that ANDRO may relieve destruction and degeneration of cartilage, we investigated its effect on growth and phenotype maintenance of rabbit articular chondrocytes* in vitro*. Examination of the cell proliferation, morphology, viability, glycosaminoglycan (GAG) synthesis, and cartilage-specific gene expression was performed. This study may provide reference for its application in treatment of OA.

## 2. Materials and Methods

### 2.1. Reagents and Instruments

We purchased 0.25% trypsin and 1% (v/v) antibiotics (penicillin 100 U/mL and streptomycin 100 U/mL) from Solarbio company in China; COL1A1(collagen I) antibody, COL2A1 (collagen II) antibody, and 3, 3-diaminobenzidine tetrahydrochloride (DAB) kit from Boster company in China; alpha-modified Eagle's medium (*α*-MEM), 20% (v/v) foetal bovine serum (FBS), dimethyl sulfoxide (DMSO), and 3-(4,5)-dimethylthiahiazo(-z-y1)-3,5-di-phenytetrazolium-romide (MTT) from Gibco company in USA; safranin O, proteinase K, Hoechst 33258, and chondroitin sulphate from Sigma company in USA; and incubator and Multiskan GO Microplate Spectrophotometer from Thermo Fisher Scientific company in USA. Other reagents and instruments in this study are as follows: the RNeasy RNA extraction kit (Tiangen Biotechnology, Beijing, China); hematoxylin-eosin (HE) kit (Jiancheng Biotech, China); reverse transcription kit (Fermentas company, USA); SYBR-Green mix kit (Roche company, Germany); FastStart Universal SYBR Green Master Mix (Roche Company, Germany); Quantitative PCR Detection System (realplex 4, Eppendorf Corporation, USA); live-dead viability assay kit (Invitrogen, USA); laser scanning confocal microscope (Nikon A1, Japan); and upright microscope (Olympus, Japan).

### 2.2. Isolation and Culture of Articular Chondrocytes

The cartilage slices were firstly harvested from 1-week-old New Zealand rabbits provided by the center of experimental animals of Guangxi Medical University with an appropriate process to attenuate the pain or discomfort of rabbits, which was approved by the Ethics Committee of Guangxi Medical University (Nanning, China; number 2014-KY-040; date: 2014-3-7). These slices primarily dissociated with 0.25% trypsin for 30 min and then with 2 mg/mL collagenase type II in *α*-MEM for 3 h. Chondrocytes were isolated through centrifugation (400 g, 5 min, 37°C) and then resuspended in *α*-MEM containing 20% (v/v) FBS and 1% (v/v) antibiotics. All cultures were maintained in 5% CO_2_ incubator at 37°C with the culture medium changed every 3 days. Cells were passaged when reaching 80–90% confluence. Confluence chondrocytes in logarithmic growth phase were prepared for further studies.

### 2.3. Preparation and Treatment of ANDRO

ANDRO was purchased from Chengdu Must Biotechnology Co., LTD. (Sichuan, China). Prior to the experiment, ANDRO was dissolved in DMSO as stock solution with the concentration of 100 mM and stored at −4°C. The stock solution of ANDRO was added to the cell culture to provide various concentrations for the following experiments. Before use, the mixed culture medium was filtered with 0.22 *μ*m filters for sterilization.

### 2.4. Cytotoxicity Assay

Articular chondrocytes were cultured in 96-well microplates pretreated with various concentrations of ANDRO (0–48 *μ*M) for 3 days. 5 mg/mL MTT was added to cultures in each well. After incubation at 37°C for 4 hours, culture medium was removed and DMSO was added (150 *μ*L per well). Microplates were gently shaken in order to obtain complete dissolving purple solution. The optical density (OD) was detected by a Multiskan GO Microplate Spectrophotometer at 570 nm.

### 2.5. Cell Proliferation Analysis and Biochemical Assay

Based on the result of cytotoxicity assay, we chose the three doses (1.5, 3, and 6 *μ*M ANDRO) with more obvious positive effect, coupled with control group (0 *μ*M ANDRO) for cell proliferation analysis and biochemical assay (also for researches below). Chondrocytes in different groups were cultured for 2, 4, and 6 days. Cells were digested by 0.25% trypsin and then resuspended in PBS containing 60 *μ*g/mL proteinase K for 16 h at 60°C. After dyeing by Hoechst 33258, proliferation of cells was tested by DNA production through ultraviolet spectrofluorometer with calf thymus DNA as a standard. The excitation wavelength is 346 nm and the emission wavelength is 460 nm. The total production of glycosaminoglycans (GAGs) was measured by absorbance value using 1,9-dimethylmethylene blue (DMMB) spectrophotometric assay at 525 nm with chondroitin sulphate as the standard sample. The synthesis and secretion of GAGs were calculated according to the standard curve and then normalized to the total DNA production, which displayed the biosynthetic activity of cells in diverse culture media.

### 2.6. Safranin O Staining

Safranin O staining was employed to further evaluate the synthesis and secretion of GAGs. Cells were fixed by 95% alcohol for 30 min and then stained with 0.1% safranin O for 10 min. After being washed by tap water, they were sealed with neutral gum. Eventually, images were captured under an upright microscope.

### 2.7. Morphological Examination

After being cultured for 2, 4, and 6 days, respectively, cells were fixed with 95% alcohol for 30 min and successively stained using HE kit. We then used an upright microscope to carry out cell morphological analysis.

### 2.8. Cell Viability Assay

Cell viability was determined by a live-dead viability assay kit. Briefly, 1 *μ*M calcein-acetoxymethyl (calcein-AM) and 1 *μ*M propidium iodide (PI) were added to cell cultures and incubated in dark for 5 min at 37°C. Images were captured utilizing a laser scanning confocal microscope.

### 2.9. Immunohistochemical Staining

Collagen type I and type II were detected by immunohistochemical staining utilizing COL1A1 (collagen I) antibody and COL2A1 (collagen II) antibody following the instructions. Cells were washed by PBS and then fixed with 95% alcohol for 30 min. Furthermore, cells were incubated with 3% H_2_O_2_ for 10 min at room temperature to remove endogenous peroxidase activity and sequentially with goat serum for 10 min at room temperature to block nonspecific staining. After primary antibodies (collagen type I and type II) were diluted to 1 : 200, they were added to wells of microplates. Fallowing it was incubation for 2 h (37°C). Second antibody and biotin labeled horseradish peroxidase were successively added for 10 min at room temperature. The chromogenic reaction of collagen type I and type II was visualized by DAB kit and counterstained with haematoxylin. Finally, cells were gradually dehydrated with graded ethanol and sealed with neutral gum. Images were captured by an upright microscope.

### 2.10. Real-Time Quantitative PCR (qRT-PCR) Analysis

To further explore the effect of ANDRO on expression of cartilage-specific genes, type I, type II, and type X collagen (COL1A1, COL2A1, and COL10A1), aggrecan, and Sox 9 mRNAs were analyzed by qRT-PCR detection. Total RNA was extracted from articular chondrocytes using the RNeasy RNA extraction kit according to the manufacturer's instruction. Approximately 300 ng of total RNA was used as a template to reversely transcribe into cDNAs using reverse transcription kit, and then cDNAs were amplified using SYBR-Green mix kit. The qRT-PCR reactions were performed by a Quantitative PCR Detection System with FastStart Universal SYBR Green Master Mix at 95°C (10 min) and 60°C (1 min). The designed primers were used for PCR as follows (Table 1) and the primer specificity was confirmed by analyzing the dissociation curve of each primer pair. Relative gene expression levels were calculated by the 2^−ΔΔCT^ method relative to glyceraldehyde-3-phosphate dehydrogenase (GAPDH) gene expression. Each gene was analyzed in triplicate to reduce randomization error.

### 2.11. Statistical Analysis

Results were expressed as mean ± SD for quantitative data. Statistical significance was determined using one way analysis of variance (ANOVA) followed by Dunnett's post hoc test. The level of significance was set to *P* < 0.05.

## 3. Results

### 3.1. Cytotoxicity Assay

**Figure 1:**
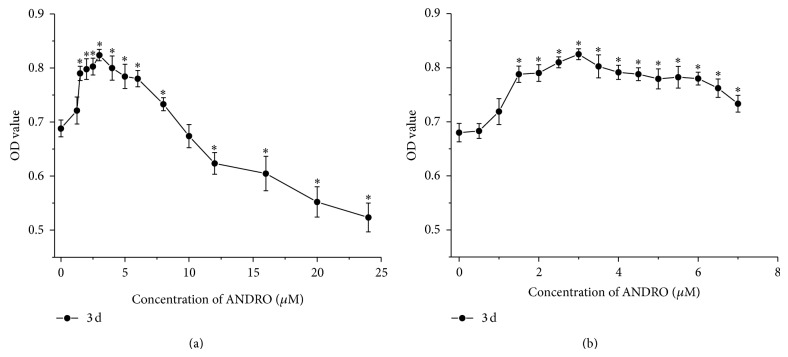
Cytotoxicity analysis of chondrocytes treated by different concentrations of ANDRO after 3 days. Results were expressed as mean ± SD (*n* = 4). ∗ indicates compared to control group, *P* < 0.05.

As shown in Figure 1, compared with the control group (0 *μ*M), 1.25–10 *μ*M ANDRO indicated low cytotoxicity. 1.5–8 *μ*M ANDRO significantly accelerated cell growth (*P* < 0.05) with the most obvious effect at the dose of 3 *μ*M. In contrast, the concentration ranging from 12 to 24 *μ*M of ANDRO showed inhibition of proliferation of rabbit articular chondrocytes* in vitro*, compared to that of control group.

### 3.2. Cell Proliferation

**Figure 2:**
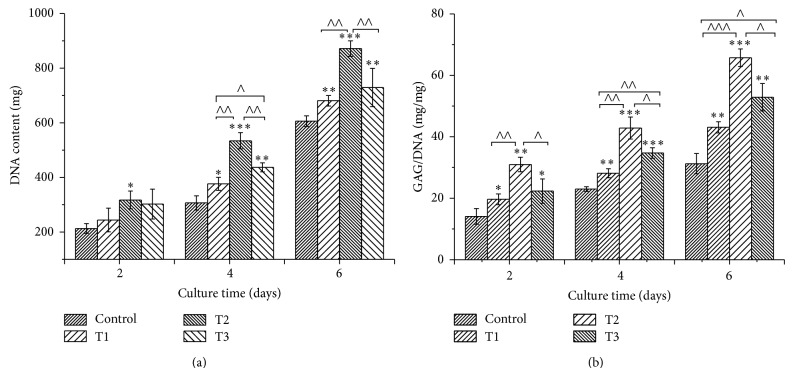
Quantification of cell proliferation by detection of DNA content and matrix production by glycosaminoglycan (GAG) analysis. (a) The proliferation of chondrocytes cultured* in vitro* with 0 (Control), 1.5 (T1), 3 (T2), and 6 *μ*M (T3) ANDRO for 2, 4, and 6 days; (b) GAG (mg) normalized to DNA (mg). Data from 3 independent experiments were evaluated, and the mean ± SD is shown. ∗, ∧ indicate *P* < 0.05; ∗∗, ∧∧ indicate *P* < 0.01;∗∗∗, ∧∧∧ indicate *P* < 0.001.

As shown in Figure 2(a), chondrocytes cultured with 1.5, 3, and 6 *μ*M ANDRO grew faster than control group (0 *μ*M ANDRO), which was proved by the significantly higher DNA content (*P* < 0.05) in the same culture period. Among the three concentrations, 3 *μ*M ANDRO exhibited the strongest promoting effect on cell growth in the same time point of culture.

### 3.3. Secretion of GAGs

As shown in Figure 2(b), there was the significantly increasing amount of GAGs provided as a ratio of GAG/DNA in ANDRO groups compared to control group at the same period (*P* < 0.05). Consistent with the cell proliferation determined by MTT assay, ANDRO at dose of 3 *μ*M indicated the best effect on GAG synthesis.

**Figure 3:**
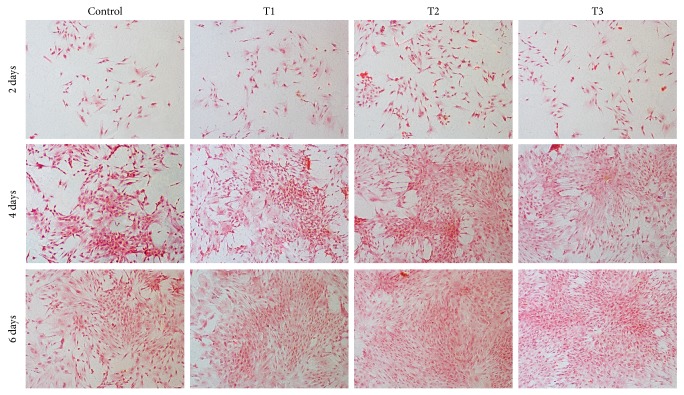
Safranin O staining showing the synthesis of extracellular matrix. Rabbit articular chondrocytes were cultured* in vitro* with 0 (Control), 1.5 (T1), 3 (T2), and 6 *μ*M (T3) ANDRO for 2, 4, and 6 days. Cell seeding density: 2 × 10^4^/mL (original magnification ×100). Scale bar = 200 *μ*m.

Figure 3 also presents deeper staining of safranin O in ANDRO groups, revealing that more cartilage-specific GAGs were produced after treatment with ANDRO. Due to the deepest staining at 3 *μ*M of ANDRO, the most proteoglycan deposition belonged in the group with ANDRO in the concentration of 3 *μ*M.

### 3.4. Cell Viability Assay

**Figure 4:**
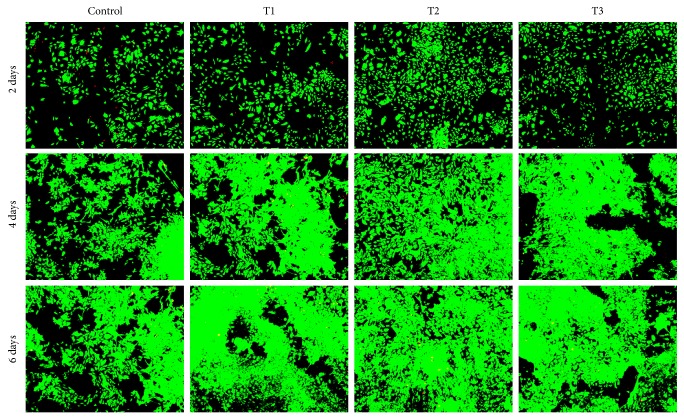
Confocal laser scanning microscopy images showing the viability of chondrocytes. Control, T1, T2, and T3 represent groups with 0 (Control), 1.5 (T1), 3 (T2), and 6 *μ*M (T3) ANDRO, respectively, to be cultured* in vitro* for 2, 4, and 6 days. Cell seeding density: 2 × 10^4^/mL (original magnification ×100). Scale bar = 200 *μ*m.

Figure 4 displays the morphology of viable and dead cells after calcein/PI staining. This determination demonstrated ANDRO groups had more viable cells and less dead ones than control group at the same time points. It revealed that ANDRO exerted positive impact on chondrocyte survival, which was accordant with the result of cell proliferation through MTT assay. Among the three ANDRO groups, the effect of ANDRO at the concentration of 3 *μ*M was better than that of others.

### 3.5. Cell Morphology

**Figure 5:**
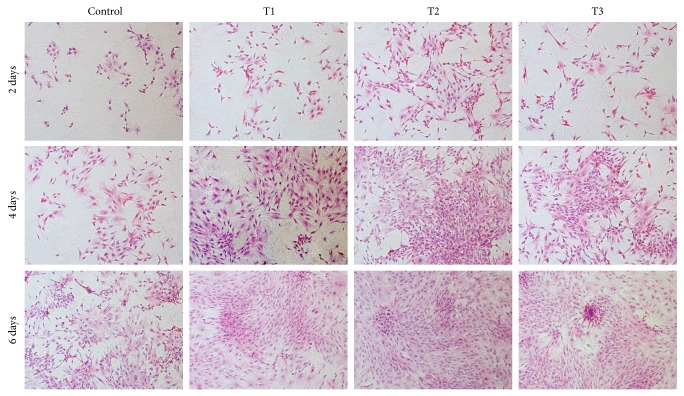
Hematoxylin-eosin staining images showing the morphology of chondrocytes. These chondrocytes were cultured* in vitro* with 0 (Control), 1.5 (T1), 3 (T2), and 6 *μ*M (T3) ANDRO for 2, 4, and 6 days. Cell seeding density: 2 × 10^4^/mL (original magnification ×100). Scale bar = 200 *μ*m.

HE staining showed that chondrocytes in ANDRO groups grew better compared with the control at the same time point (Figure 5). In ANDRO groups, we could find more round cells that represented typical morphology of chondrocytes. In addition, ANDRO at the dose of 3 *μ*M facilitated proliferation of rabbit articular chondrocytes* in vitro* more than others.

### 3.6. Secretion of Type I and Type II Collagen

**Figure 6:**
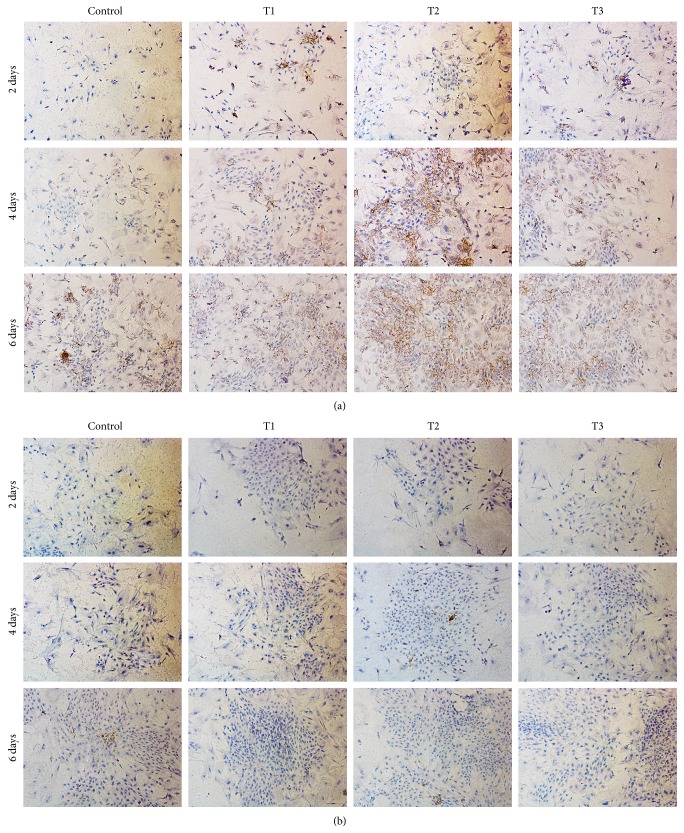
Immunohistochemical staining images revealing the presence of collagen type II (COL2A1) and type I (COL1A1). (a) The staining of collagen type II and (b) that of collagen type I. Chondrocytes were cultured* in vitro* with 0 (Control), 1.5 (T1), 3 (T2), and 6 *μ*M (T3) ANDRO for 2, 4, and 6 days. Cell seeding density: 2 × 10^4^/mL (original magnification ×100). Scale bar = 200 *μ*m.

We evaluated the deposition of matrix in rabbit articular chondrocytes* in vitro* with immunohistochemical staining of type I (COL1A1) and type II (COL2A1) collagen (Figure 6). There were more cartilage-specific type II collagen with obvious positive staining (Figure 6(a)) and less type I collagen, the indicator of chondrocyte dedifferentiation, with only very sparse and light staining (Figure 6(b)) in ANDRO groups at the same time point. By contrast, control group had less positive staining of type II collagen combined with more positive staining of type I collagen. Among the three doses of ANDRO, 3 *μ*M was superior to others in terms of maintenance of phenotype of chondrocytes.

### 3.7. Gene Expression

**Figure 7:**
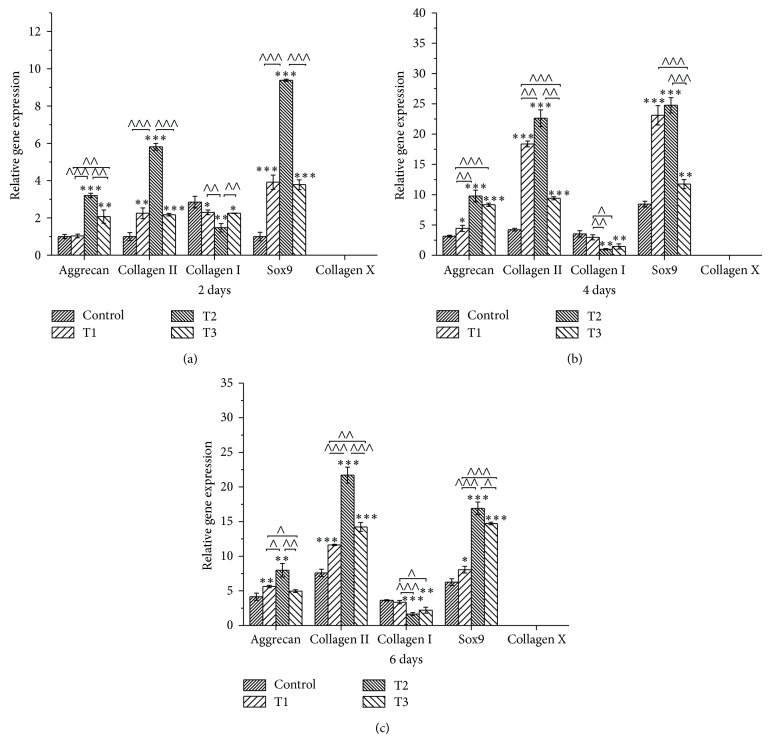
Quantitative comparison of extracellular-matrix-related gene expression by qRT-PCR. Chondrocytes were cultured with 0 (Control), 1.5 (T1), 3 (T2), and 6 *μ*M (T3) ANDRO for 2 days (a), 4 days (b), and 6 days (c) (*n* = 3 for each group at a certain time point). Gene expression levels in AND groups relative to the control group were analyzed by the 2^−ΔΔCT^ method using GAPDH as the internal control. Isoforms of collagen type I, type II, and type X studied are COL1A1, COL2A1, and COL10A1, respectively. The data reported as mean ± SD. ∗, ∧ indicate *P* < 0.05; ∗∗, ∧∧ indicate *P* < 0.01; ∗∗∗, ∧∧∧ indicate *P* < 0.001.

The positive role of ANDRO on ECM synthesis was further verified by examination of the expression of aggrecan, Sox9, and collagen type I (COL1A1), type II (COL2A1), and type X (COL10A1) (Figure 7). The expression of aggrecan, collagen II, and Sox9, the cartilage-specific genes, was observably promoted by ANDRO compared to that in the control group. In addition, the expression of collagen I gene, the marker of cell dedifferentiation, was downregulated by ANDRO compared with that of control. Furthermore, the expression of collagen X, the indicator of cell hypertrophy, could not be detectable in all groups. Among all the groups, ANDRO at the concentration of 3 *μ*M exhibited the best performance with regard to the upregulation of expression of aggrecan, collagen II, and SOX9 genes, as well as the downregulation of expression of collagen I gene.

## 4. Discussion

Diterpenes may provide antiarthritic effect to facilitate resolution of inflammation caused by cartilage lesion. Of them, ANDRO, a diterpenoid lactone mainly extracted from* Andrographis paniculata* that has been administrated for inflammation-related diseases in Asian counties with a long history, was reported to have role in treatment of arthritis [12]. Based on the hypothesis that ANDRO may relieve destruction and degeneration of cartilage, its effect on growth and phenotype maintenance of articular chondrocytes* in vitro* was investigated in this study. The results indicated that ANDRO could promote chondrocytes growth compared with control group (Figure 2). ANDRO could obviously enhance deposition of GAGs in chondrocytes (Figures 2 and 3). Proteoglycan is key part of extracellular matrices of cartilage [21], which is responsible for cartilage load-bearing capacity [22].

Consistent with the increase of deposition of GAGs in chondrocytes, ANDRO could also upregulate the expression level of genes such as Sox9, collagen II, and aggrecan (Figure 7). Chondrogenic transcription factor Sox9 is essential for the increasing level of chondrogenesis [23, 24] especially when Sox9 is coexpressed with collagen II gene [25–27]. In addition, several gene therapy approaches, say viral methods to overexpress Sox9 significantly, improved the synthesis of cartilaginous matrix produced by both bone marrow derived stem cells and articular chondrocytes [28–30]. Evidences indicated that aggrecan production was also significantly upregulated by the Sox9 gene which is an early chondrogenic marker [28, 31]. Therefore, the probable underlying mechanism is that ANDRO promoted chondrocytes growth and matrix secretion via the modulation of Sox9 expression.

Still, the result of our PCR and biochemical and immunohistochemical assay showed that the expression of collagen I which marked dedifferentiation of chondrocytes was effectively inhibited in ANDRO groups (Figures 6 and 7). Normally, chondrocytes with the differentiated phenotype could synthesize and secrete type II collagen and cartilage-specific proteoglycan that mainly comprise intercellular substance of cartilage. Dedifferentiation of chondrocytes will occur when the synthesis and secretion shift from this normal intercellular substance to an abnormal one that consists predominately of type I collagen with little proteoglycan [32–34]. Furthermore, collagen type X went undetected in ANDRO groups (Figure 7). Collagen type X is specifically associated with hypertrophic chondrocytes and omens the onset of endochondral ossification [35] and our result implies that hypertrophy of chondrocytes would not be induced by ANDRO. Therefore, the reduction of expression of collagen I and no detection of collagen X after treatment with ANDRO suggested that the dedifferentiation and hypertrophy may be prevented by ANDRO.

As for the recommendatory concentration of ANDRO, the proliferation of rabbit articular chondrocytes* in vitro* was accelerated in the range of 1.5 to 8 *μ*M ANDRO (Figure 1). At the dose of 3 *μ*M especially, ANDRO exhibited the best performance with respect to cell growth and phenotype maintenance. However, we cannot confirm whether it is suitable for articular chondrocytes of other species such as human being. Lack of experience also exists in the application of ANDRO to experiments* in vivo*. All the issues should be in need of further research.

## 5. Conclusion

ANDRO exerts positive effect on the proliferation and the phenotype maintenance of rabbit articular chondrocytes* in vitro*. ANDRO, an active principle isolated from* Andrographis paniculata*, may serve as a potential agent in the field of treatment of OA. This study may provide reference for clinical application.

## Figures and Tables

**Table 1:** Primer sequences used in qRT-PCR experiments.

mRNA	Forward primer	Reverse primer
GAPDH	5′-CTATAAATTGAGCCCGCAGC-3′	5′-ACCAAATCCGTTGACTCCG-3′
Aggrecan	5′-CTACACGCTACACCCTCGAC-3′	5′-ACGTCCTCACACCAGGAAAC-3′
Type I collagen	5′-GTTCAGCTTTGTGGACCTCCG-3′	5′-GCAGTTCTTGGTCTCGTCAC-3′
Type II collagen	5′-AAGCTGGTGAGAAGGGACTG-3′	5′-GGAAACCTCGTTCACCCCTG-3′
Type X collagen	5′-CGCTGAACGATACCAAATGCC-3′	5′-TTCCCTACAGCTGATGGTCC-3′
Sox9	5′-AAGCTCTGGAGACTTCTGAACG-3′	5′-CGTTCTTCACCGACTTCCTCC-3′
