# Expression of Concern on “Andrographolide Enhances Proliferation and Prevents Dedifferentiation of Rabbit Articular Chondrocytes: An In Vitro Study”

**DOI:** 10.1155/2021/4752718

**Published:** 2021-01-25

**Authors:** Evidence-Based Complementary and Alternative Medicine

*Evidence-Based Complementary and Alternative Medicine* would like to express concern with the article titled “Andrographolide Enhances Proliferation and Prevents Dedifferentiation of Rabbit Articular Chondrocytes: An In Vitro Study” [1]. The authors contacted the journal to say they found several errors due to the wrong placement of samples and that Figures 4 and 6 should be changed. However, they did not provide details, and the authors could not be contacted following this. We are contacting the authors' institution to request that they investigate these concerns.

In addition, the authors did not compare their findings to their related research [2].
