# Corrigendum to “Andrographolide Enhances Proliferation and Prevents Dedifferentiation of Rabbit Articular Chondrocytes: An *In Vitro* Study”

**DOI:** 10.1155/2022/9759547

**Published:** 2022-11-25

**Authors:** Li-ke Luo, Qing-jun Wei, Lei Liu, Li Zheng, Jin-min Zhao

**Affiliations:** ^1^Department of Orthopedic Trauma and Hand Surgery, The First Affiliated Hospital of Guangxi Medical University, Nanning, Guangxi 530021, China; ^2^Guangxi Key Laboratory of Regenerative Medicine, Guangxi Medical University, Nanning, Guangxi 530021, China; ^3^The Medical and Scientific Research Center, Guangxi Medical University, Nanning, Guangxi 530021, China

In the article titled “Andrographolide Enhances Proliferation and Prevents Dedifferentiation of Rabbit Articular Chondrocytes: An *In Vitro* Study” [1], the authors informed the journal that Figures 1–4 are incorrect due to errors in the placement of samples. Initially, an expression of concern was published [2], but the authors have since been able to provide the raw data and corrected figures. The corrected figures are as follows.

## Figures and Tables

**Figure 1:**
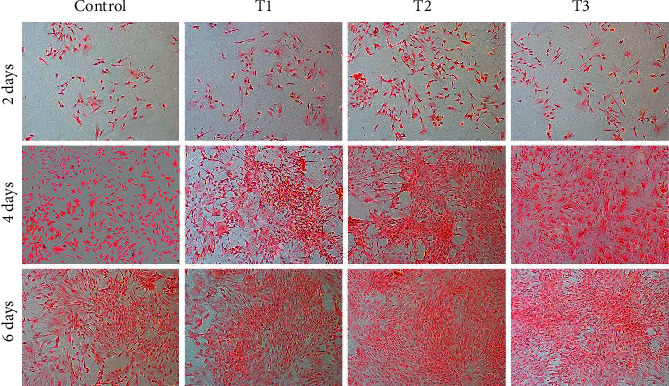
Safranin O staining showing the synthesis of extracellular matrix. Rabbit articular chondrocytes were cultured *in vitro* with 0 (Control), 1.5 (T1), 3 (T2), and 6 *μ*M (T3) ANDRO for 2, 4, and 6 days. Cell seeding density: 2 × 104/mL (original magnification ×100). Scale bar = 200 *μ*m.

**Figure 2:**
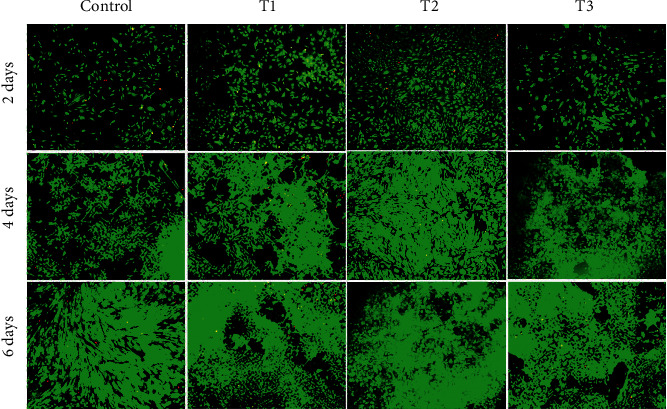
Confocal laser scanning microscopy images showing the viability of chondrocytes. Control, T1, T2, and T3 represent groups with 0 (Control), 1.5 (T1), 3 (T2), and 6 *μ*M (T3) ANDRO, respectively, to be cultured *in vitro* for 2, 4, and 6 days. Cell seeding density: 2 × 104/mL (original magnification × 100). Scale bar = 200  *μ*m.

**Figure 3:**
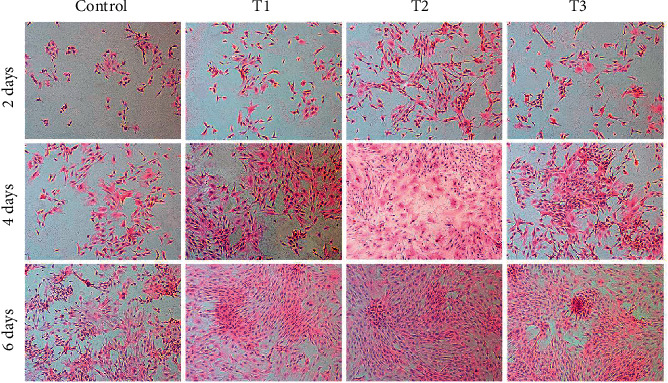
Hematoxylin-eosin staining images showing the morphology of chondrocytes. These chondrocytes were cultured *in vitro* with 0 (Control), 1.5 (T1), 3 (T2), and 6 *μ*M (T3) ANDRO for 2, 4, and 6 days. Cell seeding density: 2 × 104/mL (original magnification × 100). Scale bar = 200 *μ*m.

**Figure 4:**
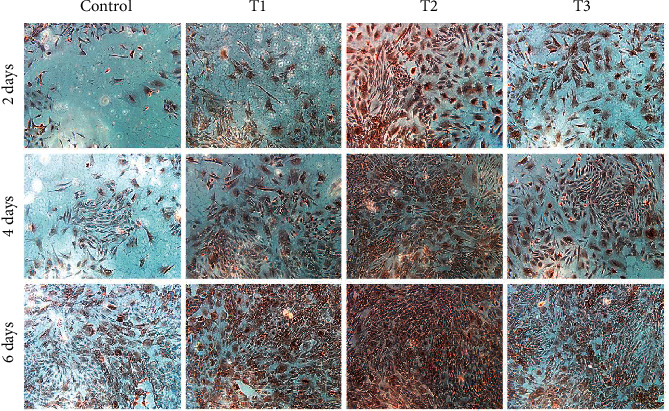
Immunohistochemical staining images revealing the presence of collagen type II (COL2A1) and type I (COL1A1). Chondrocytes were cultured *in vitro* with 0 (Control), 1.5 (T1), 3 (T2), and 6 *μ*M (T3) ANDRO for 2, 4, and 6 days. Cell seeding density: 2 × 104/mL (original magnification × 100). Scale bar = 200 *μ*m.
